# Train-the-Trainer Evaluation of Technology-Enhanced Learning in Preservice Midwifery Education in Nigeria: Qualitative Descriptive Study

**DOI:** 10.2196/93596

**Published:** 2026-08-31

**Authors:** Isabella Garti, Nadyen Shikpup, Ophelia Tonto, Michelle Gray, Halima Musa Abdul, Awwal Mohammed Ladan

**Affiliations:** 1 Faculty of Health, Charles Darwin University, 7 Ellengowan Dr, Casuaurina, Northern Territory, 0810, Australia, 1 0447252418, 1 0447252418; 2 Department of Nursing Sciences, University of Jos, Jos, Plateau State, Nigeria; 3 Ghana Health Service, Accra, Greater Accra, Ghana; 4 School of Nursing and Midwifery, University of Newcastle, Newcastle, New South Wales, Australia; 5 College of Nursing Sciences, Ahmadu Bello University, Kaduna, Kaduna State, Nigeria; 6 Department of Nursing Sciences, Bayero University, Kano, Kano State, Nigeria

**Keywords:** midwifery, midwifery educator, experiences, train the trainer, digital learning, digital technology

## Abstract

**Background:**

Midwifery education in Nigeria is undergoing a transformation in pedagogical approach. Existing traditional teaching materials are being supplemented by digital technology-enhanced learning resources; however, educators’ limited pedagogical capacity may hinder their adoption. Thus, train-the-trainer (TTT) workshops were implemented to build capacity among midwifery educators to prepare them for the implementation of a digital learning platform across 20 institutions in Nigeria.

**Objective:**

This study aimed to describe the development and qualitative evaluation of a TTT program to support the implementation of a technology-enhanced learning platform within preservice midwifery education in Nigeria. We aimed to evaluate participants’ perceptions of the training and their preparedness to implement their learning.

**Methods:**

The workshop was developed using the ADDIE (Analysis, Design, Development, Implementation, and Evaluation) instructional design model. A 3-day TTT program was delivered in 2 cohorts in July 2025 to a purposive sample of 80 institution-nominated midwifery educators and information and communication technology officers from 20 pilot institutions. A qualitative descriptive study was conducted to explore participants’ perceptions of the training and their readiness to implement and support the technology-enhanced learning platform within their institutions. Data were analyzed using qualitative content analysis.

**Results:**

Pre-workshop responses demonstrated varying levels of prior exposure to and use of digital technologies, as well as differing expectations of the training. Four themes emerged from the post-workshop evaluation. Participants perceived improvements in their understanding of pedagogical and technology-enhanced learning approaches; reported greater readiness to support implementation and knowledge transfer within their institutions; highlighted infrastructure and technical support barriers to implementation; and emphasized the need for ongoing capacity building, monitoring, and support to promote long-term sustainability.

**Conclusions:**

Rather than evaluating educational outcomes, this study provides early evidence of educator readiness, implementation considerations, and sustainability requirements for technology-enhanced learning in preservice midwifery education in low-resourced settings such as Nigeria. A multifaceted approach combining capacity building, ongoing support, technical requirements, and institutional commitment to continuous improvement is needed. Future phases of this implementation research will evaluate educational, implementation, and institutional outcomes associated with the digital learning platform.

## Introduction

Nigeria has a maternal mortality rate of 993 per 100,000 live births, more than 3 times the global average of 211 per 100,000 live births [1]. The country continues to experience poor maternal and newborn health outcomes, partly attributable to deficiencies in the quality of midwifery education. Nigeria is unlikely to make substantial progress toward achieving the Sustainable Development Goals (SDGs) related to maternal and newborn health [2] without strengthening preservice midwifery education, particularly in northern states disproportionately affected by high maternal mortality, health workforce shortages, insecurity, and ongoing unrest associated with conflict and instability [3]. Digital learning approaches can support more flexible and scalable educational delivery, strengthen educators’ capacity, and supplement existing preservice training with interactive, clinically relevant content [4-6]. Therefore, increased investment in midwifery education in Nigeria is necessary to ensure that international standards for quality education are met and to lower the extremely high maternal and newborn mortality rates [7,8].

Internationally, strengthening midwifery education is recognized as a critical strategy for reducing preventable maternal and newborn mortality and achieving the SDGs [9]. The International Confederation of Midwives (ICM) calls for midwifery education to meet international competency standards while remaining responsive to local population and health system needs [10]. Similarly, global initiatives such as the World Health Organization’s Ending Preventable Maternal Mortality agenda and the Midwifery Accelerator Framework emphasize the importance of investing in a competent, well-supported midwifery workforce as a foundation for improving maternal and newborn outcomes [11,12]. High-quality midwifery education is therefore essential for equipping practitioners with the knowledge, clinical competence, and respectful care approaches required to deliver safe and effective maternity care across diverse settings [8].

Midwifery education in Nigeria is delivered primarily through schools of nursing and midwifery, colleges of health technology, and universities, and is regulated by the Nursing and Midwifery Council of Nigeria (NMCN). The Nigerian midwifery curriculum, recently revised, does not fully align with the ICM’s Global Standards, which outline the minimum requirements for preparing competent midwives [13]. There are, however, significant challenges within preservice midwifery education in Nigeria. Midwifery training institutions face increasing pressure from workforce shortages, limited educational resources, and inconsistent access to clinical learning opportunities [8,14]. The expertise of midwifery educators in Nigeria is variable, with many tutors having strong clinical backgrounds but limited formal preparation in pedagogy, as well as limited access to contemporary teaching technologies, simulation resources, and ongoing educator development opportunities necessary to support competency-based and student-centered learning [7,14]. Thus, the delivery of education is predominantly teacher-centric, with heavy reliance on didactic instruction and rote learning [15]. Consequently, there is a growing need for innovative, student-centered educational approaches that support self-directed learning, critical thinking, clinical reasoning, and greater engagement with evidence-based midwifery practice.

Previous studies suggest that technology-enhanced learning can help address gaps in midwifery education by enriching teaching and learning and supporting more flexible, interactive, and student-centered educational approaches [6,16,17]. When integrated with traditional classroom teaching, digital technologies can accommodate diverse learning styles and promote greater learner engagement compared with predominantly didactic methods [16,18]. However, effective integration of digital technologies into midwifery curricula requires adequate educator preparation, technical support, and ongoing capacity strengthening to support implementation, adaptation, and sustainability [17,19]. As key drivers of digital transformation in education, midwifery educators require sufficient pedagogical and digital competence to effectively facilitate and support student learning in increasingly technology-enabled environments [20].

To support the effective integration and sustainability of technology-enhanced learning approaches in midwifery education, there is a need for structured capacity-building initiatives that strengthen the pedagogical and digital capabilities of educators and institutional support staff [19]. One approach increasingly used in health care education and workforce development is the train-the-trainer (TTT) model. The TTT method provides participants with the knowledge and expertise to become skilled trainers, enabling them to transfer their acquired knowledge and skills to others within their professional group. TTT models involve experts preparing a nucleus group of professionals to disseminate knowledge, skills, and evidence-based practices to their peers within their own organizations [21]. This approach supports scalable capacity strengthening and the development of local expertise, especially in limited-resource settings, promotes peer-supported learning, and may enhance the implementation of new practices within routine health care and educational settings [22]. Although TTT programs can facilitate the delivery of digital midwifery education at scale, robust and context-specific models are needed to ensure quality, sustainability, and local ownership.

Although technology-enhanced learning is increasingly being integrated into health professions education globally, the literature reports more on student learning outcomes than on the preparedness of educators and the institutional systems required to support sustainable implementation [15,23]. Limited evidence exists on how midwifery educators and institutional support staff in low- and middle-income countries experience and adapt to digital transformation initiatives, particularly within large-scale preservice education reforms [19].

Limited research has explored the design, implementation, and evaluation of training programs specifically developed to prepare midwifery educators for technology-enhanced learning approaches. Existing literature has largely focused on student experiences and digital learning outcomes, with comparatively less attention given to building the pedagogical and digital capabilities of educators responsible for integrating these approaches into preservice midwifery education. This gap is particularly evident in low- and middle-income countries, where educators may have varying levels of exposure to digital technologies, instructional design, and online facilitation strategies.

This study provides novel evidence on a needs-informed TTT approach designed specifically to prepare midwifery educators to support technology-based learning in preservice midwifery education. Its contribution lies in addressing the limited evidence on how educators in resource-constrained settings can be equipped to integrate digital platforms and digital teaching approaches into existing midwifery programs. The findings advance knowledge by identifying practical strategies to strengthen midwifery educators’ digital competencies and to support the effective and sustained implementation of technology-based learning within educational institutions. The study involved collecting baseline information on the perspectives of Nigerian midwifery educators and information and communication technology (ICT) officers regarding their participation in the initial TTT workshop.

## Methods

### Context of the Study

The NMCN digital learning platform was developed to deliver, manage, and monitor educational content for approximately 2000 preservice midwifery students across 20 pilot institutions in 11 states in Nigeria. In this pilot, 2 of the researchers, both international midwifery experts, codeveloped the digital modules. Each module features podcasts, simulations with first-person point-of-view videos, animations, and interactive technology-enhanced learning content focused on 3 maternal health conditions: postpartum hemorrhage and the E-MOTIVE bundle, multiple micronutrient supplementation for maternal anemia, and maternal sepsis. The 3 digital learning modules were developed to address major contributors to preventable maternal morbidity and mortality in Nigeria and other low-resource settings. They were designed to strengthen students’ exposure to contemporary, evidence-informed maternal health content through interactive and technology-enhanced learning approaches that could supplement existing classroom and clinical education. Midwifery students across the pilot sites will access the platform over a 12-month implementation period, during which iterative refinement of the platform and educational content will be undertaken alongside ongoing evaluation of the technology-enhanced learning intervention’s feasibility, acceptability, and usability.

### TTT Program Development and Delivery

This study is part of a broader implementation research project introducing a digital learning platform into preservice midwifery education in Nigeria. The ADDIE (Analysis, Design, Development, Implementation, and Evaluation) instructional design model guided the development of the educational products and learning resources for the TTT program. This model is used as a structured framework for instructional design [24]. The model includes analysis of learner needs and educational objectives, design and development of instructional content and delivery approaches, implementation of the training, and evaluation to inform ongoing refinement and improvement. Although presented in phases, the ADDIE model is iterative and nonlinear in practice. As part of the evaluation phase, a qualitative descriptive study was conducted to explore the perspectives and initial experiences of midwifery educators and institutional ICT officers who participated in the TTT workshop.

### The ADDIE Framework

#### Analysis

The analysis phase identified gaps in preservice midwifery education in Nigeria, including reliance on predominantly teacher-centered approaches that did not adequately support student learning outcomes or meet the objectives of the NMCN. A baseline assessment conducted between September and December 2024 identified shortages of midwifery educators, limitations in digital learning infrastructure and skills laboratories, and competency gaps among educators, preceptors, and final-year students in key maternal health areas, including postpartum hemorrhage management. These findings highlighted the need for strengthened educator capacity, improved learning resources, and innovative teaching approaches, including refresher training and technology-enhanced learning strategies.

#### Design

The design phase focused on planning the implementation of a technology-enhanced learning platform within existing preservice midwifery programs. Learning needs assessments identified limited digital literacy, insufficient experience with learner-centered teaching approaches, and varying levels of familiarity with digital technologies among educators. The TTT approach was selected to support scalable implementation and sustainability across pilot institutions. Educational content and module topics were predetermined within the broader project and developed collaboratively by Nigerian midwifery academics, project partners, and key stakeholders, including the NMCN, to ensure contextual relevance and alignment with curriculum standards. Two 3-day workshop sessions were designed for separate participant cohorts. Training objectives, session content, instructional activities, logistical requirements, and facilitation strategies were developed during this phase. The workshops incorporated practical activities, case studies, small-group discussions, and adult learning principles to promote active participation and peer learning. Nigerian midwifery educators led the delivery of the training, supported by the project team and technology partners, to enhance contextual appropriateness and communication.

#### Development

During the development phase, the original program developers created technology-enhanced learning resources and instructional materials for the TTT workshops, as they had the most in-depth understanding of the program. Due to contextual factors, including language and local communication styles, it was not feasible for the program developers to deliver the training directly. Therefore, it was planned that experienced Nigerian midwifery faculty with in-depth knowledge of the program would facilitate the training, with ongoing support and guidance from the original developers. A combination of theoretical and practical learning was used to equip trainers with the competencies needed to deliver high-quality cascade training. Workshop materials were designed to model learner-centered and interactive teaching approaches while supporting participants’ engagement with adult learning principles and instructional design concepts. A training handbook was developed to provide guidance on curriculum alignment, lesson planning, and integration of digital technologies into teaching and assessment. Additional workshop activities, including group-based exercises and interactive digital tools such as Mentimeter, were incorporated to support collaborative learning and participant engagement. Technology partners also developed platform-specific instructional resources covering platform architecture, navigation, login procedures, backend functionality, and user management. Effective adaptation strategies were introduced during the initial training and supported by planned supplementary training to reinforce learning and build confidence. Session schedules, durations, and training logistics were finalized during this phase (Multimedia Appendix 1).

#### Implementation

The TTT workshops were delivered in Abuja between July 14, 2025, and July 19, 2025, in 2 cohorts through intensive 3-day training sessions. The workshops aimed to prepare participants to support the implementation of the digital learning platform within their institutions and to cascade training to other midwifery educators. Training combined hands-on workshops facilitated by the technology partner with in-person and virtual sessions delivered by Nigerian educators and supported by the original program developers and the project team. Educational strategies included presentations, facilitated discussions, group work, demonstrations, and practical activities. Participants received an overview of the Moodle platform architecture and engaged in guided and independent navigation exercises exploring platform functionality, user roles, and educational content. Training explored challenging areas in greater depth, such as the integration of digital learning, and incorporated dedicated opportunities for questions and shared problem-solving. This initial training session focused on foundational digital literacy and platform engagement, with future training planned to support participants in independently creating and uploading digital learning resources and integrating the platform into existing teaching approaches.

#### Evaluation

This evaluation was not designed to formally assess feasibility, but rather to explore participants’ perceptions and experiences of the TTT workshops to inform ongoing refinement of the training program and broader implementation processes. The evaluation used anonymous qualitative surveys administered before and after the training workshops. Pre-training questions explored participants’ prior experiences, expectations, and perceptions of digital technologies, while post-training questions examined the perceived usefulness and acceptability of the training, as well as participants’ perceived readiness to support implementation within their institutions. These insights were intended to inform educator readiness, engagement, and acceptability, while guiding future refinement, sustainability, and cascading of the intervention.

### Research Design

A qualitative descriptive design [25] was used, as it enables researchers to remain close to the data while providing a comprehensive summary of participants’ perspectives and experiences without aiming to generate theory or explore deeper, lived, or cultural meanings [26]. Qualitative description is particularly suited to examining who, what, and the context of events or experiences and is useful for exploring poorly understood phenomena through the accounts of participants [25]. This approach was selected because little is currently known about the perspectives and experiences of midwifery educators in Nigeria regarding the introduction of technology-enhanced learning resources and the expectations associated with adopting and disseminating new digital approaches within preservice midwifery education. Consequently, a qualitative descriptive methodology was considered appropriate for exploring participants’ perceptions, experiences, and responses to this educational change introduced by the NMCN. Findings from the evaluation were used to identify areas requiring additional support, refinement, and future capacity-building activities.

### Participants and Sampling

Participants were purposively selected by the NMCN from participating institutions based on their teaching responsibilities in courses linked to the digital learning modules, as course allocations are predetermined for the academic year. Institutional ICT officers were also included due to their roles in supporting digital infrastructure and platform implementation within the schools. Eligible participants included seasoned midwifery educators and ICT officers attending the TTT workshops who were not on leave, were involved in the relevant courses, and were willing to participate in the qualitative evaluation conducted before and after the training. A total of 80 participants attended 2 separate 3-day workshops. Although participants were nominated by their institutions to attend the training, participation in the qualitative evaluation component was voluntary, and participants were informed that their decision to participate or decline would not affect their involvement in the training program.

### Data Collection

Qualitative data were collected through anonymous, written, open-ended responses before and after the workshops. This qualitative method can generate meaningful and rich insights, even when individual responses are brief, and minimizes participant discomfort [27]. This approach was selected based on participant preference, as some attendees were uncomfortable with audio-recorded interviews or group discussions, and it enabled participants to share their perspectives in their own words while minimizing burden within the intensive training schedule. Surveys were completed individually at the venue. Participants could remain at their allocated seats or choose another suitable location within the premises and were given approximately 30 minutes to record their responses. They wrote their responses by hand on the paper-based surveys and could request extra sheets as needed.

The pre-training survey comprised 5 questions that assessed participants’ prior experience, expectations, and concerns about the training (Multimedia Appendix 2). The post-training survey included 9 open-ended questions that evaluated participant satisfaction, key learnings, intended actions, and areas requiring further training to inform future program planning (Multimedia Appendix 3).

Despite the anonymous, written format, social desirability bias may still have influenced responses, with participants potentially feeling inclined to provide favorable feedback due to their institutional nomination to attend the training and the broader implementation context.

### Data Analysis

The research team followed the systematic steps of qualitative content analysis as described by Erlingsson and Brysiewicz [28], which include familiarization with the data, identifying meaning units, condensing those units, coding, categorizing, and formulating themes. Content analysis was chosen because it provides a systematic and transparent method for analyzing open-ended qualitative data across multiple participants. Data were analyzed manually without the use of qualitative software. Manual coding was selected to support collaborative analysis across the international research team and ensure accessibility for all researchers involved in the project. Two researchers independently reviewed and coded the responses before meeting to compare interpretations and discuss coding differences. Any discrepancies were resolved through discussion and consensus within the broader research team. Meaningful data segments relevant to the study objectives, including perceptions of the training, perceived learning gains, challenges, and anticipated application of skills, were identified and coded. The research team met regularly via Zoom to discuss coding decisions, resolve discrepancies through consensus, merge overlapping codes, and refine categories. Related codes were grouped into broader descriptive categories, which were subsequently developed into higher-level themes. Throughout the analytic process, categories and themes were repeatedly reviewed against the original written responses to ensure they accurately reflected participants’ perspectives and experiences. Given the qualitative descriptive nature of the study and the use of brief written responses, the analysis aimed to achieve sufficient informational richness relevant to the study objectives rather than formal theoretical data saturation associated with interpretive methodologies.

### Trustworthiness

To enhance the trustworthiness of the findings, several strategies were used throughout the analysis [29]. Credibility was enhanced through independent coding by members of the research team, followed by consensus discussions to resolve differences in interpretation and refine the coding framework. Dependability was supported through consistent data collection and analysis procedures, and by documenting coding decisions as the analysis progressed. To ensure confirmability, regular team meetings were held in which interpretations were critically examined and grounded in participants’ responses rather than in individual assumptions. Transferability was facilitated by providing detailed information about the participants, training, and the study context. Researcher reflexivity was embedded throughout the analytic process and involved ongoing consideration of the researchers’ roles, assumptions, and involvement in the program, as well as their potential influence on data collection, analysis, and interpretation.

### Ethical Considerations

Ethical approval for this study was obtained from the Human Research Ethics Committee of Charles Darwin University, Australia (ethics number H25071), and the institutional Research Ethics Committee at the Liverpool School of Tropical Medicine (ethics number 24‐074). The study was conducted in accordance with internationally accepted ethical principles for research involving human participants. All participants were provided with adequate information about the study objectives, procedures, potential risks, and benefits. Informed consent was obtained from all participants before data collection. Consent was sought both verbally and in writing, and participation was not compulsory. Confidentiality and anonymity were maintained throughout the study, and participation was entirely voluntary, with the right to withdraw at any stage without penalty. Prior to data collection, participants were informed verbally and in writing that completion of the evaluation activities was optional and would not affect their participation in the training or their professional roles. None of the participants objected to participating. Participants were also informed that they could decline to answer any question or withdraw at any time without consequence.

## Results

In total, 80 participants took part in the study, comprising 60 female participants and 20 male participants. Most participants (n=58, 72.5%) were midwifery educators, alongside 21 ICT officers and 1 clinical instructor (Table 1).

**Table 1.** Participant characteristics.

Variable and category	Values, n (%)
Gender	
Male	20 (25)
Female	60 (75)
Participant role	
Midwifery educator	58 (72.5)
ICT^a^ officer	21 (26.3)
Clinical instructor	1 (1.2)
Participating states	
Yobe	8 (10)
Katsina	8 (10)
Kaduna	8 (10)
Kano	8 (10)
Niger	8 (10)
Plateau	8 (10)
Borno	8 (10)
Ebonyi	8 (10)
Bauchi	8 (10)
Zamfara	4 (5)
Akwa-Ibom	4 (5)
Total, N	80 (100)

a ICT: information and communication technology.

### Pre-Training Responses

Pre-training responses revealed considerable variation in participants’ prior exposure to digital tools, current technology use, and expectations for the training. Although some participants reported prior digital training, these experiences were often described as basic, outdated, or insufficient, whereas others relied mainly on self-directed learning and trial-and-error. This finding suggests limited access to structured and ongoing digital capacity development across participating institutions.

Participants’ current use of digital tools was generally limited to basic applications, such as PowerPoint, WhatsApp, and online videos, with only a few having prior experience with platforms like Moodle. Despite these limitations, participants expressed strong interest in developing practical digital teaching skills, particularly in navigating learning management systems, creating digital content, integrating multimedia resources, improving online student engagement, and enhancing digital assessment practices. The findings indicate a strong demand for hands-on, practice-oriented training to strengthen educators’ readiness for technology-enhanced teaching.

### Post-Training Responses

#### Post-Training Perceptions and Experiences

The post-training responses highlighted 4 themes that articulated participants’ perceptions and experiences of the TTT session—theme 1: developing pedagogical and digital teaching competencies; theme 2: self-perceived confidence and readiness to support implementation; theme 3: perceived barriers and logistical challenges; and theme 4: sustaining technology-enhanced learning.

#### Theme 1: Developing Pedagogical and Digital Teaching Competencies

Following the training workshop, participants described a greater understanding of both educational pedagogy and digital concepts. They described acquiring foundational knowledge and practical skills to effectively use digital tools and navigate the teaching platform. This highlights that the training was instrumental in helping educators align digital resources with the existing curriculum, thereby strengthening the relevance and integration of technology within learning activities. The structured and progressive nature of the training appeared to help participants connect pedagogy, curriculum outcomes, and technology-enhanced learning in more practical ways.

*The training was progressive, with each part building upon the previous one. It was a step-by-step process: you must understand the learning objectives in the curriculum because they go hand in hand*.

[Cohort 2, Respondent 23]

The lesson-planning component was particularly valued because it strengthened participants’ understanding of learning outcomes, learner characteristics, and Bloom’s taxonomy, while improving their ability to design effective lessons and organize courses sequentially.

*I have also learned how to make a lesson plan, which will bring a breakthrough in my teaching activities and help me achieve the outcomes I want for my students. It [Lesson plan] also helps us, as educators, to create a sequential model that makes our work flexible and enhances student learning*.

[Cohort 1, Respondent 1]

Participants further noted that the knowledge gained from lesson planning and course organization would improve student learning outcomes.

*This will improve learning outcomes for my students..., understanding how a course is designed and organized online*.

[Cohort 2, Respondent 22]

Participants described gaining a broader understanding of educational principles, including different types of learners, teaching approaches, learning activities, and Bloom’s taxonomy. They perceived this knowledge as enhancing their understanding of how teaching and learning can be effectively supported through the learning management system.

*I also gained knowledge on the different types of students, lecturers, and educators, the various kinds of learning activities, and the six types of Bloom’s taxonomy. All these have helped me understand teaching and learning through the LMS.*

[Cohort 1, Respondent 4]

Participants reported gaining practical skills in using the learning management system, including creating assessments, delivering lectures, uploading educational content, developing multimedia learning materials, and monitoring student engagement and participation.

*I have gained a great deal from learning how to log in to the computer and navigate it. I also learned how to use the computer in teaching and incorporating videos. In addition, I gained knowledge of the learning management system, how to navigate the portal, and an overview of creating content*.

[Cohort 1, Respondent 15]

Participants described the content and resources as appropriate, useful, and accessible, supporting both teaching and student engagement. Participants appreciated the use of Nigerian voiceovers and actors, which increased the realism and contextual relevance of the learning experience.

*The parts of the training I found most useful and impactful were those that made teaching and learning more realistic, efficient, and effective by utilizing podcasts, videos, pictures, animations, drag-and-drop features, and scenarios. This is because these will make teaching and learning easier and more interesting for both the trainer and the learner*.

[Cohort 2, Respondent 23]

These findings suggest that participants required support not only in the technical use of digital platforms but also in the pedagogical foundations necessary to effectively integrate technology-enhanced learning into existing teaching practices. The overlap between educational and digital competencies highlights that successful digital transformation requires more than technological infrastructure; it also requires educator preparedness and confidence in applying learner-centered teaching approaches.

#### Theme 2: Self-Perceived Confidence and Readiness to Support Implementation

Following the training, participants described greater perceived confidence and readiness to support the implementation of technology-enhanced learning within their institutions. Participants attributed this readiness to the practical, hands-on nature of the training, which enabled them to actively engage with the platform, explore its functionality, and apply newly acquired knowledge in a supportive learning environment. There were 2 subthemes: valuing practical, hands-on learning and commitment to training others.

##### Subtheme 1: Valuing Practical, Hands-On Learning

Participants consistently highlighted the workshop’s practical, interactive design as a key facilitator of learning. Opportunities to navigate the platform, engage with activities, and apply concepts in real time were perceived as particularly valuable for building familiarity with technology-enhanced learning approaches.

*I found the training to be very engaging and participatory, with everyone actively involved typing and clicking on their computers, logging in and out*.

[Cohort 2, Respondent 3, post-training]

Another participant added:

*The practical aspect of the training was the most useful because it made learning easier for me. It was better to do it myself, and I felt more confident by the second day. It has motivated me to use a laptop to teach. Henceforth, I will continue to try my hand at creating blended lectures*.

[Cohort 2, Respondent 4, post-training]

The workbook and workshops enhanced participants’ confidence and motivation, equipping educators with resources to support students’ use of digital tools during implementation.

*I was previously hesitant about using technology in training and teaching, but this program gave me the confidence to utilize digital tools effectively*.

[Cohort 2, Respondent 5, post-training]

However, some participants felt that additional training sessions would help consolidate learning and build confidence over time. They noted that the breadth of content introduced during the workshop required additional time and practice to fully understand and apply it. They collectively emphasized that ongoing training and support would be essential to sustain their engagement.

*Organise training 2‐3 times per year...especially creating PowerPoints*.

[Cohort 1, Respondent 20, post-training]

*I suggest that more days should be allocated to this type of training next time, enabling us to fully grasp everything*.

[Cohort 2, Respondent 20, post-training]

These findings indicate the importance of embedding continuous professional development, technical support, and implementation coaching within technology-enhanced learning initiatives to facilitate long-term adoption and integration into teaching practice.

##### Subtheme 2: Commitment to Training Others

Participants expressed a strong willingness to disseminate the knowledge they acquired through this process through step-down training, viewing this as essential to ensuring the platform’s success and sustainability. Many described intentions to organize training sessions for colleagues, support the implementation of the digital learning platform, and begin developing or uploading their own educational content.

*I plan to train other educators [on this training], involve students, and ensure that they can access the platform. I will be very excited to start uploading my own modules to improve the learning and teaching process*.

[Cohort 1, Respondent 5]

Participants also outlined strategies for preparing to cascade the training, including reviewing workshop materials, revisiting the training workbook and notes, and developing action plans tailored to their institutional contexts.

*This is how I plan to apply what I learned in my own teaching practice: first, to become very familiar with the process of logging in and using the platform so that I can effectively transfer the knowledge and skills to my colleagues and students. I will then ensure that my students are referred to the modules. I will also create content to further support their learning*.

[Cohort 2, Respondent 3]

Another participant explained,

*Before I train my colleagues, I will review my notes and develop an action plan*.

[Cohort 2, Respondent 18]

These findings suggest that participants viewed themselves not only as recipients of training but also as potential change agents within their institutions. Their willingness to train colleagues, develop educational content, and plan implementation activities reflects their perceived readiness to support the broader adoption of technology-enhanced learning.

### Theme 3: Perceived Barriers and Logistical Challenges

Participants identified infrastructure and ongoing support as critical determinants of successful implementation. Common concerns included unreliable internet connectivity, unstable power supply, limited sensitization, and access to timely technical support. The provision of free internet access was frequently proposed, underscoring the need to address systemic and infrastructural barriers to the sustainable integration of technology-enhanced learning.

*All necessary facilities, such as internet access and power supply, should be ensured, as they are very important*.

[Cohort 2, Respondent 17]

Participants identified technical support and access to adequate resources as essential for sustaining digital learning and the effective implementation of the program. Participants also emphasized that resource availability, including access to laptops and embedded training materials, could enhance the consistency and ease of training delivery.

*Yes, my suggestion is that laptops should be provided to master trainers, with all the topics embedded into the laptop*.

[Cohort 2, Respondent 20]

Participants highlighted the importance of collaboration between educators and ICT officers, emphasizing the need for shared training opportunities and equal access to the platform. This finding suggests that successful implementation of technology-enhanced learning requires a team-based approach, with clearly defined roles and shared responsibility to support sustainability and reduce reliance on individual staff members.

*Both midwife educators and Information Technology officers should be trained at the same level so that the ICT officers will not be overwhelmed with loads of responsibilities*.

[Cohort 1, Respondent 5]

### Theme 4: Sustaining Technology-Enhanced Learning

Participants highlighted the importance of continuity in future training activities, expressing a preference for the same facilitators who introduced the platform to lead subsequent training sessions, thereby providing continuity in learning, reinforcing key concepts, and supporting a deeper understanding of both the platform and its educational applications.

*There is a need for more training on content creation and uploads. The NMCN should organise such training two to three times per year*.

[Cohort 2, Respondent 29]

*Future training should include the same educators who introduced the platform to ensure continuity and a deeper understanding*.

[Cohort 1, Respondent 8]

Participants identified several strategies to support successful implementation and ongoing engagement with the platform. These included regular system maintenance and updates to enhance usability, development of user-friendly navigation tutorials such as YouTube videos, routine check-ins to monitor progress and address challenges, and the establishment of clear expectations for appropriate behavior in both classroom and online learning environments.

*a) Regular maintenance and updates that can enhance user experience, b) tutorials on navigating the platform, which can be in the form of YouTube videos, c) implementation of regular check-in strategies, and d) clear guidelines for acceptable behaviour within the classroom and online*.

[Cohort 1, Respondent 22]

Participants generally valued the training but suggested that online sessions could be made more engaging through greater visual interaction with facilitators. This finding highlights the importance of facilitator presence in online learning environments and reflects the practical challenges of delivering technology-enhanced learning in settings where limited internet bandwidth may restrict the use of video and other interactive features.

*I suggest that the online lectures be made more engaging and less boring, perhaps by allowing us to see the presenters or facilitators*.

[Cohort 2, Respondent 26]

Together, these findings suggest that sustained implementation requires ongoing capacity strengthening, resource investment, and responsive support systems, rather than one-off training interventions.

## Discussion

### Principal Findings

Participants in this study perceived that the TTT program enhanced their understanding of technology-enhanced learning and increased their confidence in supporting its implementation in preservice midwifery education. They also expressed a willingness to champion digital learning in their institutions. However, participants consistently identified inadequate digital infrastructure, limited technical support, and the need for ongoing training as important considerations for sustaining its implementation. The study’s insights contribute to a growing evidence base by identifying contextual factors that educators perceive as important for integrating digital learning into preservice midwifery education in resource-constrained settings.

The findings suggest that the success of TTT programs for technology-enhanced learning extends beyond knowledge acquisition and requires attention to implementation readiness, contextual barriers, and strategies that support long-term sustainability, consistent with evidence synthesized in a recent scoping review [19]. Such evidence is scarce in midwifery education, particularly in the context of nationwide initiatives in countries with high maternal mortality, where strengthening the midwifery workforce is critical to improving maternal and newborn outcomes [9].

Prior studies have demonstrated that TTT initiatives can improve knowledge, confidence, and teaching competencies [21,30,31]. Although this study did not measure these outcomes quantitatively, participants reported increased confidence, readiness to train others, and motivation to apply newly acquired skills, all of which are required for successful implementation and sustainability [30,32]. In contexts where educator shortages and limited resources constrain professional development opportunities, such gains may be critical for supporting the successful implementation and scale-up of technology-enhanced learning. These qualitative findings extend the existing evidence by providing a deeper understanding of the processes through which TTT programs build implementation capacity and foster sustainable educational change.

Gaps in pedagogical and digital competencies can directly affect the quality, implementation, and sustainability of technology-enhanced learning, as well as training outcomes at different levels. Although participants were enthusiastic about the TTT program and eager to apply their new knowledge, many lacked a foundational understanding of teaching and learning principles, reflecting a reliance on traditional didactic approaches. These findings should be understood within the broader Nigerian educational environment, where educator shortages, limited professional development opportunities, and inadequate digital skills training are well-documented [7,15]. Within midwifery education, large student cohorts and high educator-to-student ratios further reinforce didactic teaching practices [15]. In this context, the program addressed an important capacity gap by strengthening educators’ pedagogical and digital competencies and equipping them to support teaching and learning despite significant workforce and resource constraints.

In this study, resistance to educational innovation was not the issue. Participants demonstrated openness to change and a willingness to experiment with new teaching approaches. While participants were enthusiastic about technology-enhanced learning, they frequently highlighted unreliable internet connectivity and electricity supply as key barriers to implementation. These infrastructure challenges were viewed as important considerations for successful implementation, as reported in other settings [6,16,33,34], and are also consistent with broader reports describing digital infrastructure limitations across Nigeria [35,36]. While planned national investments in fiber-optic infrastructure may improve future connectivity, these findings suggest that digital education initiatives must be accompanied by investments in reliable internet connectivity, digital infrastructure, devices, and ongoing technical support that enable educators and students to fully engage with these resources to achieve their intended impact [34].

Sustaining digital learning initiatives requires supportive policy, leadership commitment, and ongoing training [34]. In low- and middle-income countries, where resources are constrained and priorities shift, understanding sustainability is critical [37]. Given Nigeria’s size and security challenges, technology-enhanced learning strategies may offer a viable and scalable solution. Notably, there is a strong political commitment to advancing midwifery in Nigeria, as reflected in the Nigeria Strategic Directions for Nursing and Midwifery (NSDNM) 2025 to 2030, which acknowledges ongoing gaps in training, deployment, and retention of skilled midwives [38]. Situating participant-identified barriers within this national reform agenda highlights that, while targeted training to build educator capacity is essential, sustainable improvement will require continued investment in educational infrastructure, workforce development, and supportive policy frameworks.

The findings have several practical implications. First, digital capacity-building programs should incorporate both technological skills and foundational pedagogical training to support meaningful educational transformation. Second, implementation strategies should include ongoing mentorship, technical support, and opportunities for peer learning to reinforce skills gained during training [34]. This also includes diffusing expertise within the system through a strong community of practice, which was set up via WhatsApp group chats in this case. Third, policymakers and educational leaders should recognize that investments in educator capacity alone are unlikely to achieve sustainable change without concurrent improvements in digital infrastructure, internet access, and institutional support systems [16].

Overall, the findings suggest that the TTT approach is a feasible and practical strategy for implementing technology-enhanced learning at scale. Future studies should examine the longer-term impact of the TTT model on behavioral outcomes, including educator practice, institutional adoption of technology-enhanced learning, and student learning outcomes. Evaluation of the planned training cascade will be particularly important in determining whether knowledge and skills are successfully transferred to additional educators and sustained over time. Further studies should also explore factors that influence implementation across different institutional and geographical contexts and identify strategies that support long-term sustainability of digital education initiatives in resource-constrained settings.

### Limitations

This study has several limitations. First, a quantitative assessment of participants’ pre-training and post-training knowledge and skills was not conducted, as the training was intentionally designed to foster a supportive, nonthreatening learning environment. Second, although a paper-based open-ended survey was deemed appropriate, it may have limited opportunities for deeper exploration of individual experiences and perspectives. Third, participants were nominated by their institutions to attend the training, which may have influenced perceptions of voluntariness, despite the evaluation being optional and anonymous. As the findings were based on self-reported written feedback collected within a funded implementation project, socially desirable responses cannot be excluded. Moreover, we did not objectively assess knowledge, skills, competence, or the extent of change attributable to the program. Therefore, the findings reflect perceived readiness, usefulness, and acceptability rather than measurable improvements in teaching practice or digital competence. In addition, the short duration of the training may have limited participants’ confidence to cascade the training, and the absence of a structured buddying approach may have reduced opportunities for peer support and ongoing capacity building. As the broader implementation project is ongoing, future phases of the study will evaluate longer-term educational, institutional, and implementation outcomes associated with the technology-enhanced learning platform.

### Conclusions

This study evaluated participants’ perceptions of a needs-informed TTT program and their preparedness to implement a technology-enhanced learning platform in preservice midwifery education in Nigeria. It provides novel evidence in an underresearched area by showing how targeted educator training can strengthen digital competencies and support the implementation and sustained use of technology-based learning. Rather than evaluating educational outcomes or changes in practice, this study provides early evidence of educators’ readiness to adopt student-centered innovative learning resources. Participants are motivated to share their learning and are enthusiastic about adopting the new resources on a digital learning platform. Nevertheless, our findings suggest that national implementation may require a structured strategy to equip educators with pedagogical principles to advance the widespread application across Nigeria. The findings also indicate that expanding technology-enhanced learning in preservice midwifery education in Nigeria is likely to require sustained institutional and government commitment to strengthening the infrastructure required to support digital learning platforms.

## Supplementary material

10.2196/93596 Multimedia Appendix 1 Summary of the 3-day train-the-trainer workshop.

10.2196/93596 Multimedia Appendix 2 Pre-training open-ended questions.

10.2196/93596 Multimedia Appendix 3 Post-training open-ended questions.

## Abbreviations

ADDIE: Analysis, Design, Development, Implementation, and Evaluation
ICM: International Confederation of Midwives
ICT: information and communication technology
NMCN: Nursing and Midwifery Council of Nigeria
NSDNM: Nigeria Strategic Directions for Nursing and Midwifery
SDG: Sustainable Development Goal
TTT: train-the-trainer
