# Supplementary material for: Train-the-Trainer Evaluation of Technology-Enhanced Learning in Preservice Midwifery Education in Nigeria: Qualitative Descriptive Study
Source: JMIR Nurs. 2026 Aug 31;9:e93596. doi: 10.2196/93596 (PMC13529257; doi:10.2196/93596)
Supplement: Supplementary file 1 [file nursing-v9-e93596-s001.docx]

**Supplementary Table 1: Summary of the 3 -day Train-the -Trainer’s Workshop**

| Day | Session | Mode | Duration |
| --- | --- | --- | --- |
| 1 | Opening remarks, participants introduction and expectations | In-person | 45 minutes |
|  | Objectives of training and Expectations | In-person | 15 minutes |
|  | Overview of the Digital Learning Initiative and Portal Goals, Platform Architecture and User Interface Overview | In-person | 45 minutes |
|  | Hands-on Portal Walkthrough: Dashboard, Navigation, User Roles & Account Setup (Admin, Educator, Student) | In-person Hands-on | 2 Hours |
|  | Practise Sessions by Participants | In-person hands-on | 1Hour 30 minutes |
|  | Troubleshooting, User Access Issues, and Offline Access Option | In-person | 45 minutes |
|  | Q&A, Feedback Collection, and Close of Day 1 | In-person | 20 minutes |
|  |  |  |  |
| 2 | Recap of previous day activities, Overview of objectives, icebreaker activity | In-person | 10 minutes |
|  | Curriculum Design & Learning Outcomes  Key principles, writing and understanding learning outcomes, and aligning with frameworks  Learner and teacher types | In-person | 1 Hour 30 minutes |
|  | Writing Learning Objectives  Using Bloom’s Taxonomy, SMART objectives, Bloom’s levels, practice activity | In-person | 2 Hours |
|  | Lesson Planning & Learning Activities  Content planning and structuring, Templates | Zoom-based Virtual session | 1 Hour 30minutes |
|  | Introduction to E-learning & Moodle Course Setup  E-learning concepts, Navigating Moodle, Uploading files | Virtual via zoom | 1 Hour |
|  |  |  |  |
| 3 | Recap of previous day activities, Overview of objectives, icebreaker activity | In-person | 10 minutes |
|  | PPH Module run through | In-person | 1 Hour 35 minutes |
|  | Multimedia & Interactive Learning  Instructional design elements, writing for digital, integrating different content types: video/audio, and H5P activity.  OER, Adapting traditional formats | Zoom-based virtual session | 1 Hour 30 Minutes |
|  | Evaluation & Collaboration  Managing and organising content  Using Moodle tools to assess students, IT collaboration, and wrap-up | Zoom-based virtual session | 1 Hour 30 minutes |
|  | Recap/ Questions | In-person | 1 Hour 15 minutes |
