# Supplementary material for: Train-the-Trainer Evaluation of Technology-Enhanced Learning in Preservice Midwifery Education in Nigeria: Qualitative Descriptive Study
Source: JMIR Nurs. 2026 Aug 31;9:e93596. doi: 10.2196/93596 (PMC13529257; doi:10.2196/93596)
Supplement: Supplementary file 2 [file nursing-v9-e93596-s002.docx]

**Pre-Training Open-ended questions**

Midwifery Faculty’s Expectations about Training

1. Please describe any previous training or support on the use of digital tools in midwifery education.
2. Describe your current experience using digital tools or digital content in your teaching.
3. What are your expectations about participating in this digital teaching and learning training based on the information you received?
4. Please share any concerns you have about participating in this digital teaching and learning training.
5. What skills or knowledge would you like to gain from this training on using digital content
