# Supplementary material for: Train-the-Trainer Evaluation of Technology-Enhanced Learning in Preservice Midwifery Education in Nigeria: Qualitative Descriptive Study
Source: JMIR Nurs. 2026 Aug 31;9:e93596. doi: 10.2196/93596 (PMC13529257; doi:10.2196/93596)
Supplement: Supplementary file 3 [file nursing-v9-e93596-s003.docx]

**Post-Training Open-ended Questions**

1. What new skills or knowledge did you gain from this training on using digital content in teaching midwifery?
2. Which part(s) of the training did you find most useful or impactful, and why?
3. What teaching or learning needs did this training help address for you?
4. Which aspects of the training did you find less relevant or unnecessary? Please explain.
5. How would you describe your experience of participation and engagement during the training sessions?
6. How do you plan to apply what you learned in your own teaching practice?
7. How would you evaluate the quality of the digital tools and resources presented during the training?
8. What aspects of the training did you find most useful, and were there any areas you felt could be improved or further strengthened?
9. Do you have any suggestions for improving the training in future sessions?
